# Comparison of *Scheffersomyces stipitis* strains CBS 5773 and CBS 6054 with regard to their xylose metabolism: implications for xylose fermentation

**DOI:** 10.1002/mbo3.5

**Published:** 2012-03

**Authors:** Stefan Krahulec, Regina Kratzer, Karin Longus, Bernd Nidetzky

**Affiliations:** Institute of Biotechnology and Biochemical Engineering, Graz University of TechnologyPetersgasse 12/I A-8010, Graz Austria

**Keywords:** CBS 5773, CBS 6054, ethanol, *Pichia stipitis*, *Scheffersomyces stipitis*, xylose

## Abstract

The various strains of *Scheffersomyces stipitis* (*Pichia stipitis*) differ substantially with respect to their ability to ferment xylose into ethanol. Two *P. stipitis* strains CBS 5773 and CBS 6054 have been most often used in literature but comparison of their performance in xylose fermentation under identical conditions has not been reported so far. Conversion of xylose (22 g/L) by each of these *P. stipitis* strain was analyzed under anaerobic and microaerobic conditions. Ethanol yields of ∼0.41 g/g were independent of strain and conditions used. Glycerol and acetate were formed in constant yields of 0.006 g/g and 0.02 g/g, respectively. Xylitol formation decreased from ∼0.08 g/g to ∼0.05 g/g upon switch from anaerobic to microaerobic conditions. Specific activities of enzymes of the two-step oxidoreductive xylose conversion pathway (xylose reductase and xylitol dehydrogenase) matched for both strains within limits of error. When xylose was offered at 76 g/L under microaerobic reaction conditions, ethanol yields were still high (0.37–0.39 g/g) for both strains even though the xylitol yields (0.12–0.13 g/g) were increased as compared to the conditions of low xylose concentration. *P. stipitis* strains CBS 5773 and CBS 6054 are therefore identical by the criteria selected and show useful performance during conversion of xylose into ethanol, irrespective of the supply of oxygen.

## Introduction

*Scheffersomyces stipitis (Pichia stipitis)* is an ascomycetous yeast that has become widely known for its ability to rapidly ferment xylose, the major pentose in nature, into ethanol (Hahn-Hägerdal et al. 2006; Jeffries et al. 2007; Agbogbo and Coward-Kelly 2008, 2008; Jeffries and Van Vleet 2009; for general reviews: see Girio et al. 2010). In prior work, it has been demonstrated that the fermentation behavior differs widely among different *P. stipitis* strains used and that it is also strongly dependent on the cultivation conditions (Dellweg et al. 1984; Ferreira et al. 2011). An early study by Dellweg et al. (1984) showed variation in *Y*_ethanol_ (ethanol yield) and *Y*_xylitol_ (xylitol yield) in the range 0.32–0.40 g/g and 0.05–0.19 g/g, respectively, when four strains of *P. stipitis* (CBS 5773, 5774, 5775, 5776) were compared in anaerobic conversion experiments (30 g/L xylose, pH 5.0). Two different strains of *P. stipitis* have most often been used in literature (for review, see Agbogbo and Coward-Kelly, 2008), namely strain CBS 5773 (IFO1687, NBRC1687, ATCC58376, NRRL Y-7124) and strain CBS 6054 (IFO10063, ATCC58785, NRRL Y-11545). For strain CBS 5773, *Y*_ethanol_ was reported to increase from 0.24 to 0.49 g/g in dependence of a decrease in the initial xylose concentration from 50 to 5 g/L. *Y*_xylitol_ decreased from 0.20 to 0.05 g/g in response to the same change in xylose concentration (Dellweg et al. 1984). On the contrary, anaerobic conversion of xylose (50 g/L; pH 5.5) with strain CBS 6054 did not give detectable amounts of xylitol while *Y*_ethanol_ was only 0.25 g/g (Skoog and Hahn-Hägerdal 1990), suggesting that strain CBS 6054 could differ substantially from strain CBS 5773 with respect to its capability of xylose fermentation.

There has recently been much renewed interest in the use of *P. stipitis* for fermentation of xylose and even scale-up studies were undertaken. Different substrates were used (Sanchez et al. 2002; Agbogbo et al. 2006; Agbogbo and Wenger 2007; Bajwa et al. 2009; Diaz et al. 2009) and also different process conditions were applied (Agbogbo et al. 2007; Fu et al. 2009; Lee et al. 2009; 2011a, 2011b; Silva et al. 2010; Li et al. 2011). A systematic comparison of *P. stipitis* CBS 5773 and CBS 6054 therefore seemed to be of high interest, supporting the wealth of applied studies on xylose fermentation by this organism.

*Pichia stipitis* CBS 5773 and CBS 6054 are furthermore of interest because the xylose pathway from these two strains was the preferred point of departure for construction of xylose-fermenting strains of *Saccharomyces cerevisiae* that in its natural form cannot utilize xylose (Chu and Lee 2007; Hahn-Hägerdal et al. 2007a, 2007b; Matsushika et al. 2009). Metabolic utilization of xylose by *P. stipitis* occurs via a two-step oxidoreductive pathway that is common among xylose-utilizing yeasts and consists of xylose reductase (*Ps*XR) and xylitol dehydrogenase (*Ps*XDH). The metabolic engineering strategy pursued was to insert the genes encoding *Ps*XR and *Ps*XDH and co-express them with the endogenous gene for xylulose kinase, thus establishing a pathway for efficient conversion of xylose into xylulose 5-phosphate. However, the resulting *S. cerevisiae* strains differed significantly in their performance during sugar fermentation, the distribution of products obtained from xylose varied in a broad range (Table S1). Interestingly, the evidence in Table S1 could be interpreted to imply that yeast strains harboring the xylose pathway from *P. stipitis* CBS 5773 produce less xylitol (*Y*_xylitol_ ≤ 0.10 g/g xylose) than the corresponding strains developed from *P. stipitis* CBS 6054 for which *Y*_xylitol_ could increase to very high values of up to 0.59 g/g xylose.

The reaction conditions used with both *P. stipitis* strains in literature span a wide range, making it difficult to compare the reported results. There is therefore the clear need to carry out strain comparison under exactly identical conditions. This study was performed to resolve complexity arising from the different *P. stipitis* strains used in xylose conversions experiments with the native yeast as well as regarding the source of genes for construction of recombinant *S. cerevisiae* strains.

## Materials and Methods

### Strains and media

*Scheffersomyces stipitis* CBS 5773 and CBS 6054 were kind gifts from Dr. Marko Kuijper (BIRD Engineeering, HG Schiedam, The Netherlands). Mineral media were used as described elsewhere (Krahulec et al. 2010), except that KH_2_PO_4_ was applied at 14.4 g/L. Note that complex media were not tested because determination of a carbon balance would have been difficult in that case. An initial pH of 6.5 was used, and all cultures were supplemented with 22 or 76 g/L xylose. Aerobic precultures grown on xylose (30°C, 130 rpm) were harvested at an OD_600_ of ∼6.

### Xylose conversion

Xylose conversions were carried out in duplicates at 30°C either in the complete absence of air oxygen or microaerobically. Anaerobic conversions were done at 180 rpm in 100-mL round-bottom flasks that were tightly sealed with a septum and an aluminum cap. A photograph in Figure S1 shows the flask and the sealing used. The sealing fulfilled requirements of the Hungate technique for work under anaerobic conditions. The flasks were never opened during the conversion, and the overpressure generated during the reaction certainly ensured that during sampling (through the septum) not a significant amount of O_2_ entered the medium.

Microaerobic conversions (110 rpm) were carried out in 300-mL baffled shake flasks that were closed with a rubber stopper. A glass tube equipped with a closed flexible tube and a needle was inserted into the rubber stopper, allowing a small oxygen transfer rate of about ∼7 μM/h measured experimentally. Figure S1 shows a photograph of the fully equipped flask. All cultures were carefully purged with N_2_ before and after inoculation. Initial cell densities were between OD_600_ ∼1.4 (anaerobic) and OD_600_ ∼1.6 (microaerobic) for reactions using 22 g/L xylose. When instead 76 g/L xylose was used, the OD_600_ was ∼6. Samples were taken at suitable times and cell growth was recorded as increase in OD_600_, validated by cell dry weight (CDW) measurements after 0, 75, and 145 h of xylose conversion. Work-up of the samples (Krahulec et al. 2010) and HPLC analysis of external metabolites (Petschacher and Nidetzky 2008) were according to reported protocols. Carbon recovery was calculated assuming that 1 mol CO_2_ was formed per mole of acetate or ethanol produced. Formation of biomass and production of pyruvate (< 0.001 g/g xylose) were taken into account. Biomass was assumed to have a carbon content of 43%, as determined for *S. cerevisiae* (Lange and Heijnen 2001).

### Enzyme activity measurements

Cells were harvested after 145 h by centrifugation and disrupted by vortex mixing (10 times for 45 sec) with 0.5-mm glass beads. The ratio of cell wet weight (g), glass beads (g), and potassium phosphate buffer (mL) (50 mM; pH 7.0) was 1:1:2. Cell debris was removed by centrifugation. Enzyme activities and protein concentrations were determined in the crude extract as described in Krahulec et al. (2009). XR activity was measured in 50-mM potassium phosphate buffer pH 7.0 using 670-mM xylose and 0.3-mM NADH. XDH activity was measured in 50-mM TRIS/HCl buffer pH 9.0 using 140-mM xylitol and 2-mM NAD^+^.

## Results and Discussion

**Figure 1:**
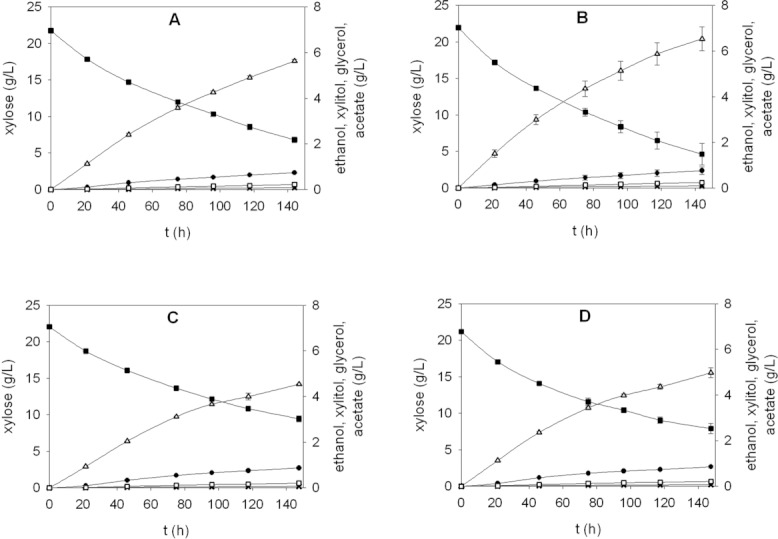
Time-course analysis of xylose conversion under microaerobic (A, B) and anaerobic (C, D) reaction conditions. Panels (A, C) and (B, D) show results for *Pichia stipitis* CBS 5773 and CBS 6054, respectively. The symbols indicate xylose (full squares), ethanol (triangles), xylitol (circles), acetate (empty squares), and glycerol (stars). Microaerobic conversions were inoculated to a starting OD_600_ of ∼1.6, while the initial OD_600_ in anaerobic conversions was ∼1.4. Slightly higher xylose conversion indicated for CBS 6054 is due to a comparably higher increase in cell density observed for CBS 6054. Note that differences in *μ*_max_ (see Table 1) are in the range of the experimental error.

The measured time courses of conversion of xylose (22 g/L) by each of the two strains under the different conditions used are displayed in Figure 1. Yield coefficients and specific growth rates calculated from xylose conversions are summarized in Table 1. Specific activities of *Ps*XR (EC 1.1.1.307) and *Ps*XDH (EC 1.1.1.9) recorded from yeast cell extracts are also shown in Table 1. Total carbon recoveries indicate that less than 10% of the total carbon from xylose remained unaccounted for. Carbon recoveries below 100% might partially be caused by a true CO_2_ yield that is slightly higher than the one estimated from the production of acetate and ethanol. Carbon flux through the oxidative pentose phosphate pathway is known to be high in *P. stipitis* when glucose is used as substrate (Fiaux et al. 2003). Assuming this might also be the case when xylose is utilized, it could mean that more CO_2_ is generated than is accounted for in the calculation.

**Table 1:** Comparison of xylose fermentation by *Pichia stipitis* CBS 5773 and CBS 6054 using a substrate concentration of 22 g/L

	Shake flask (microaerobic)		Closed flask (anaerobic)	
		
	CBS 5773	CBS 6054	CBS 5773	CBS 6054
*μ*_max_ (1/h)	0.008 ± 0.001	0.013 ± 0.005	0.008 ± 0.001	0.012 ± 0.001
*q*_xylose_ (g/g CDW/h)^1^	0.25 ± 0.04	0.22 ± 0.02	0.25 ± 0.03	0.27 ± 0.04
*Y*_ethanol_ (g/g)^2^	0.41 ± 0.01	0.41 ± 0.01	0.40 ± 0.01	0.42 ± 0.01
*Y*_xylitol_ (g/g)^2^	0.056 ± 0.004	0.051 ± 0.020	0.084 ± 0.003	0.078 ± 0.001
*Y*_glycerol_ (g/g)^2^	0.006 ± 0.001	0.006 ± 0.001	0.006 ± 0.001	0.006 ± 0.001
*Y*_acetate_ (g/g)^2^	0.018 ± 0.001	0.018 ± 0.003	0.020 ± 0.001	0.020 ± 0.001
C-recovery (%)	90	92	96	100
XR (μmol/min/mg)	0.66 ± 0.06	0.81 ± 0.02	0.54 ± 0.05	0.59 ± 0.10
XDH (μmol/min/mg)	2.9 ± 0.2	2.8 ± 0.1	1.9 ± 0.3	2.4 ± 0.4

^*^Xylose uptake rates are snapshots after 22 h of fermentation. Note that uptake rates decreased during incubation times. Cell dry weight was determined in duplicates after 0, 75, and 145 h.

†Yield coefficients are given in g/g xylose and were calculated from a dataset for which a linear relationship between metabolite production and xylose consumption was confirmed.

Table 1 provides a useful set of data with which to compare the two *P. stipitis* strains among each other and with other strains described in literature. Growth on xylose was very low for each strain under complete exclusion of air oxygen but also under the microaerobic conditions. The xylose uptake rates (*q*_xylose_) of the *P. stipitis* strains are considered to be high, approaching the upper limit of reported *q*_xylose_ values for xylose-fermenting strains of *S. cerevisiae*. Auxiliary modifications (e.g., overexpression of nonoxidative pentose phosphate pathway, evolutionary engineering) of recombinant *S. cerevisiae* strains were usually necessary to reach *q*_xylose_ values >0.2 g CDW/g/h (Matsushika et al. 2009). *Y*_ethanol_ was also high, and remarkably, its value of ∼0.41 g/g was not sensitive to oxygen availability under the applied conditions. The *Y*_ethanol_ measured herein for *P. stipitis* CBS 5773 is in good agreement with literature data on the same strain (Dellweg et al. 1984; Delgenes et al. 1986). Using oxygen-limited reaction conditions in continuous culture whereby the oxygen transfer rate was defined as lower than 1 mM/h, Skoog and Hahn-Hägerdal (1990) found that *Y*_ethanol_ for *P. stipitis* CBS 6054 went up to values of 0.48 g/g. Under completely anoxic reaction conditions, however, these authors observed a drop of *Y*_ethanol_ to a value of 0.25 g/g that is at variance with our observations (Table 1). Note however that differences in *Y*_ethanol_ might be caused by the different reaction conditions used, as already suggested by Dellweg et al. (1984). We further show in Table 1 that *Y*_glycerol_ and *Y*_acetate_ were identical for both strains and did not alter significantly between microaerobic and anaerobic conversions. The anaerobic *Y*_xylitol_ of 0.08 g/g for strain CBS 5773 is in accordance with literature data (Dellweg et al. 1984). Switch from anaerobic to microaerobic conditions went along with a ∼33% decrease in *Y*_xylitol_ for both strains, presumably reflecting enhancement of the NAD^+^ regeneration ability of the yeast cells under conditions of controlled oxygen supply. In spite of the conflicting evidence on the role of oxygen availability on *Y*_ethanol_ (Dellweg et al. 1984; Delgenes et al. 1986;Skoog and Hahn-Hägerdal 1990), reports agree on the notion that low aeration results in enhancement of the specific ethanol production rate (*q*_ethanol_). Under the microaerobic conditions used here where the oxygen transfer rate was much smaller than in previous studies, a dependence of *q*_ethanol_ on oxygen availability was not significant with limits of experimental error. The observed increase in final ethanol titer by about 30% upon switch from complete oxygen exclusion to microaeration is at least partially caused by different initial cell densities (see Fig. 1). Further investigation of the dependence of ethanol production parameters (*q*_ethanol_, *Y*_ethanol_, final ethanol titer) on oxygen transfer rate was however beyond the scope of this study.

**Figure 2:**
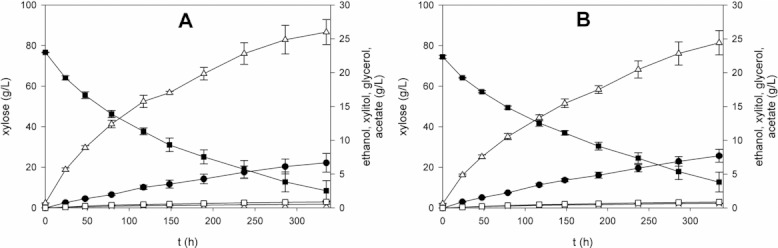
Effect of elevated substrate concentration (76 g/L) on xylose conversion under microaerobic reaction conditions by *Pichia stipitis* CBS 5773 (A) and CBS 6054 (B). The symbols indicate xylose (full squares), ethanol (triangles), xylitol (circles), acetate (empty squares), and glycerol (stars). A starting OD_600_ of ∼6 was used.

Considering that the substrate concentration used in the first round of our experiments was certainly too low to be of a technological interest, we decided to perform additional conversions at a 3.5-fold elevated concentration of xylose. Microaerobic conditions were chosen. Figure 2 summarizes the results, showing that the xylose was consumed efficiently by both strains. Physiological parameters determined from the time courses of substrate utilization and products formation are given in Table 2. *Y*_ethanol_ was still high for both strains even though *Y*_xylitol_ was increased as compared to the analogous (microaerobic) experiment done with a xylose concentration of 22 g/L. *Y*_glycerol_ and *Y*_acetate_ were very similar at high and low xylose concentration. Switch in substrate level from 22 to 76 g/L resulted in a slightly lowered *q*_xylose_. It could therefore be worth to examine a possible dependence of *q*_xylose_ on the xylose concentration in future experiments. Like at 22 g/L, growth was hardly significant at the elevated xylose concentration. Comparison of the results in Tables 1 and 2 reveals that both strains continued to show a highly similar performance in xylose fermentation when the substrate level was raised from 22 to 76 g/L.

**Table 2:** Comparison of xylose fermentation by *Pichia stipitis* CBS 5773 and CBS 6054 under microaerobic reaction conditions using an elevated substrate concentration of 76 g/L

	CBS 5773	CBS 6054
*μ*_max_ (1/h)	0.007 ± 0.001	0.006 ± 0.002
*q*_xylose_ (g/g CDW/h)^1^	0.18 ± 0.004	0.15 ± 0.015
*Y*_ethanol_ (g/g)^2^	0.37 ± 0.001	0.39 ± 0.005
*Y*_xylitol_ (g/g)^2^	0.127 ± 0.023	0.118 ± 0.011
*Y*_glycerol_ (g/g)^2^	0.008 ± 0.001	0.011 ± 0.001
*Y*_acetate_ (g/g)^2^	0.013 ± 0.001	0.015 ± 0.001
C-recovery (%)	89	93

^*^Xylose uptake rates are snapshots after 22 h of fermentation. Cell dry weight was determined in duplicates after 0, 75, and 145 h.

†Yield coefficients are given in g/g xylose and were calculated from a dataset for which a linear relationship between metabolite production and xylose consumption was confirmed.

In line with the comparable fermentation behavior of the two strains, the specific XR and XDH activities in cell extracts of the two strains were in the same range (Table 1). It is remarkable that contrary to the low *Y*_xylitol_ for both native strains of *P. stipites* (Tables 1 and 2), the corresponding recombinant strains of *S. cerevisiae* often produced large amounts of xylitol (Table S1). Although *Y*_xylitol_ is a complex parameter derived from multiple physiological effects, there is general agreement that one main reason for xylitol production is incomplete recycling of NAD(P)H coenzyme in the steps of *Ps*XR and *Ps*XDH (Jeffries 2006; Chu and Lee 2007; Hahn-Hägerdal et al. 2007a, 2007b). While *Ps*XDH is dependent on NAD^+^ and does not use NADP^+^, *Ps*XR utilizes both NADPH and NADH with clear preference for NADPH (Chu and Lee 2007, and references therein). It was confirmed through different approaches of protein and metabolic engineering that decrease in the redox imbalance generated at the level of *Ps*XR–*Ps*XDH results in lowering of *Y*_xylitol_ (for a recent review, see Matsushika et al. 2009). However, different coenzyme specificities of XR from two *P. stipitis* strains can be ruled out since it is known from literature that the primary structures of *Ps*XR in strain CBS 5773 (Amore et al. 1991) and CBS 6054 (Hallborn et al. 1991) as well as the coding genes (CBS 5773: X59465.1; CBS 6054: NC_009045.1) are identical.

Summarizing, *P. stipitis* strains CBS 5773 and CBS 6054 were examined in xylose-to-ethanol fermentations under completely anoxic and also microaerobic reaction conditions. The two strains showed identical performance among each other within limits of the experimental error under all conditions used. This identity notwithstanding, the importance of the work lies in the systematic and detailed comparison of the two prominent strains under exactly identical cultivation conditions, providing relevant evidence previously not available in the literature. Each *P. stipitis* strain converted xylose into ethanol in an excellent yield of up to 0.41 g/g while formation of xylitol and other by-products was minimal (22 g/L xylose) or low (76 g/L xylose). Of note, this highly favorable distribution of fermentation products from xylose remains to be achieved in genetically engineered strains of *S. cerevisiae* in which the xylose pathway from *P. stipitis* is expressed heterologously (Matsushika et al. 2009). Our results therefore reinforce the notion that the two native *P. stipitis* strains should be regarded as promising candidates for use in xylose conversion (Agbogbo and Coward-Kelly 2008; Li et al. 2011). The ongoing studies with known or newly isolated strains of *P. stipitis* are thus strongly supported (Sanchez et al. 2002; Agbogbo et al. 2006; Agbogbo and Wenger 2007; Bajwa et al., 2009; Diaz et al. 2009; Fu et al. 2009; Lee et al. 2009; 2011a, 2011b; Li et al. 2011). Findings reported herein are also clear in showing that low-level supply of oxygen is not a requirement for achieving a high ethanol yield from xylose using *P. stipitis*, settling the disquiet about conflicting observations that have been made in the past (Dellweg et al. 1984; Delgenes et al. 1986; Skoog and Hahn-Hägerdal 1990; Silva et al. 2010).

This study is of additional interest in the context of metabolic engineering of *S. cerevisiae* for xylose fermentation. The evidence presented does not support a role of an *external factor*, specific for the particular *P. stipitis* strain used as source of the xylose pathway, in determining differences in the xylose fermentation capabilities of the recombinant strains of *S. cerevisiae*. It is much more likely that there are *intrinsic factors* of variety in play, and these probably pertain to the used parental strains of *S. cerevisiae* as well as to the genetic strategies applied for heterologous gene expression. The levels of intracellular activity of *Ps*XR and *Ps*XDH in the recombinant *S. cerevisiae* strains might be revealing. However, a rigorous comparison would necessitate constancy of conditions used in the analysis that is unfortunately lacking in the published data.

## Supporting information

**Table S1.** Comparison of xylose-fermenting strains of *Saccharomyces* harboring genes encoding *Ps*XR and *Ps*XDH from *Pichia stipitis* CBS 5773 and CBS 6054.

**Figure S1.** Photograph showing the flasks used for anaerobic (*left*) and microaerobic (*right*) conversions of xylose.
