# Supplementary material for: Comparison of Scheffersomyces stipitis strains CBS 5773 and CBS 6054 with regard to their xylose metabolism: implications for xylose fermentation
Source: Microbiologyopen. 2012 Mar;1(1):64–70. doi: 10.1002/mbo3.5 (PMC3426399; doi:10.1002/mbo3.5)
Supplement: Supplementary file 1 [file mbo30001-0064-SD1.pdf]

Table S1: Comparison of xylose-fermenting strains of *Saccharomyces* harboring genes encoding *Ps*XR and *Ps*XDH from *P. stipitis* CBS 5773 and CBS 6054.

Strain	XR XDH from CBS	*Saccharomyces* background	Initial xylose conc. (g/L)	$Y_{ethanol}$ (g/g)	$Y_{xylitol}$ (g/g)	Ref.
1400(pLNH32)	5773	1400[d]	80	0.34	0.06	(1)
1400(pLNH32)	5773	1400[d]	50	0.3	0.08	(2)
MA-N4	5773	INVSc1	45	0.34	0.10	(3)
MA-R4	5773	IR-2	45	0.35	0.04	(4)
MA-D4	5773	D452-2	45	0.35	0.08	(4)
MA-T4	5773	Type-II	45	0.34	0.09	(4)
H158-pXks[a]	6054	GPY55-15α	80	0.22	0	(5)
H158-pXks[b]	6054	GPY55-15α	80	0.27	0.03	(5)
CEN.PK-pXks[a]	6054	CenPK2-1C	80	0.16	0.25	(5)
CEN.PK-pXks[b]	6054	CenPK2-1C	80	0.21	0.36	(5)
TMB3001	6054	CenPK 113-7A	50	0.31	0.29	(6)
TMB3001	6054	CenPK 113-7A	10	0.44	0.21	(7)
TMB3260[c]	6054	CenPK 113-7A	50	0.30	0.13	(8)
TMB3250	6054	ENY.WA-1A	50	0.30	0.30	(6)
TMB3399	6054	USM21	20	0.05	0.59	(9)

All strains additionally provide overexpression of the endogenous gene for xylulose kinase. The Table is an update of the summary given by (10).

[a] in defined media; [b] in complex media; [c] overproduces XR; [d] Fusion between *Saccharomyces diastaticus* and *Saccharomyces uvarum*

References

1. **Moniruzzaman, M., Dien, B. S., Skory, C. D., Chen, Z. D., Hespell, R. B., Ho, N. W. Y., Dale, B. E., and Bothast, R. J.:** Fermentation of corn fibre sugars by an engineered xylose utilizing *Saccharomyces* yeast strain, World J. Microbiol. Biotechnol., **13**, 341-346 (1997).

2. **Ho, N. W., Chen, Z., and Brainard, A. P.:** Genetically engineered *Saccharomyces* yeast capable of effective cofermentation of glucose and xylose, Appl. Environ. Microbiol., **64**, 1852-1859 (1998).

3. **Matsushika, A., Watanabe, S., Kodaki, T., Makino, K., Inoue, H., Murakami, K., Takimura, O., and Sawayama, S.:** Expression of protein engineered NADP+-dependent xylitol dehydrogenase increases ethanol production from xylose in recombinant *Saccharomyces cerevisiae*, Appl. Microbiol. Biotechnol., **81**, 243-255 (2008).

4. **Matsushika, A., Inoue, H., Murakami, K., Takimura, O., and Sawayama, S.:** Bioethanol production performance of five recombinant strains of laboratory and industrial xylose-fermenting *Saccharomyces cerevisiae*, Bioresour. Technol., **100**, 2392-2398 (2009).

5. **Johansson, B., Christensson, C., Hobley, T., and Hahn-Hägerdal, B.:** Xylulokinase overexpression in two strains of *Saccharomyces cerevisiae* also expressing xylose reductase and xylitol dehydrogenase and its effect on fermentation of xylose and lignocellulosic hydrolysate, Appl. Environ. Microbiol., **67**, 4249-4255 (2001).

6. **Jeppsson, M., Johansson, B., Hahn-Hägerdal, B., and Gorwa-Grauslund, M. F.:** Reduced oxidative pentose phosphate pathway flux in recombinant xylose-utilizing *Saccharomyces cerevisiae* strains improves the ethanol yield from xylose, Appl. Environ. Microbiol., **68**, 1604-1609 (2002).

7. **Träff-Bjerre, K. L., Jeppsson, M., Hahn-Hägerdal, B., and Gorwa-Grauslund, M. F.:** Endogenous NADPH-dependent aldose reductase activity influences product formation during xylose consumption in recombinant *Saccharomyces cerevisiae*, Yeast, **21**, 141-150 (2004).

8. **Jeppsson, M., Träff, K., Johansson, B., Hahn-Hägerdal, B., and Gorwa-Grauslund, M. F.:** Effect of enhanced xylose reductase activity on xylose consumption and product distribution in xylose-fermenting recombinant *Saccharomyces cerevisiae*, FEMS Yeast Res., **3**, 167-175 (2003).

9. **Wahlbom, C. F., van Zyl, W. H., Jönsson, L. J., Hahn-Hägerdal, B., and Otero, R. R.:** Generation of the improved recombinant xylose-utilizing *Saccharomyces cerevisiae* TMB 3400 by random mutagenesis and physiological comparison with *Pichia stipitis* CBS 6054, FEMS Yeast Res., **3**, 319-326 (2003).

10. **Chu, B. C. and Lee, H.:** Genetic improvement of *Saccharomyces cerevisiae* for xylose fermentation, Biotechnol. Adv., **25**, 425-441 (2007).
