# Supplementary material for: Comparison of Scheffersomyces stipitis strains CBS 5773 and CBS 6054 with regard to their xylose metabolism: implications for xylose fermentation
Source: Microbiologyopen. 2012 Mar;1(1):64–70. doi: 10.1002/mbo3.5 (PMC3426399; doi:10.1002/mbo3.5)
Supplement: Supplementary file 2 [file mbo30001-0064-SD2.doc]

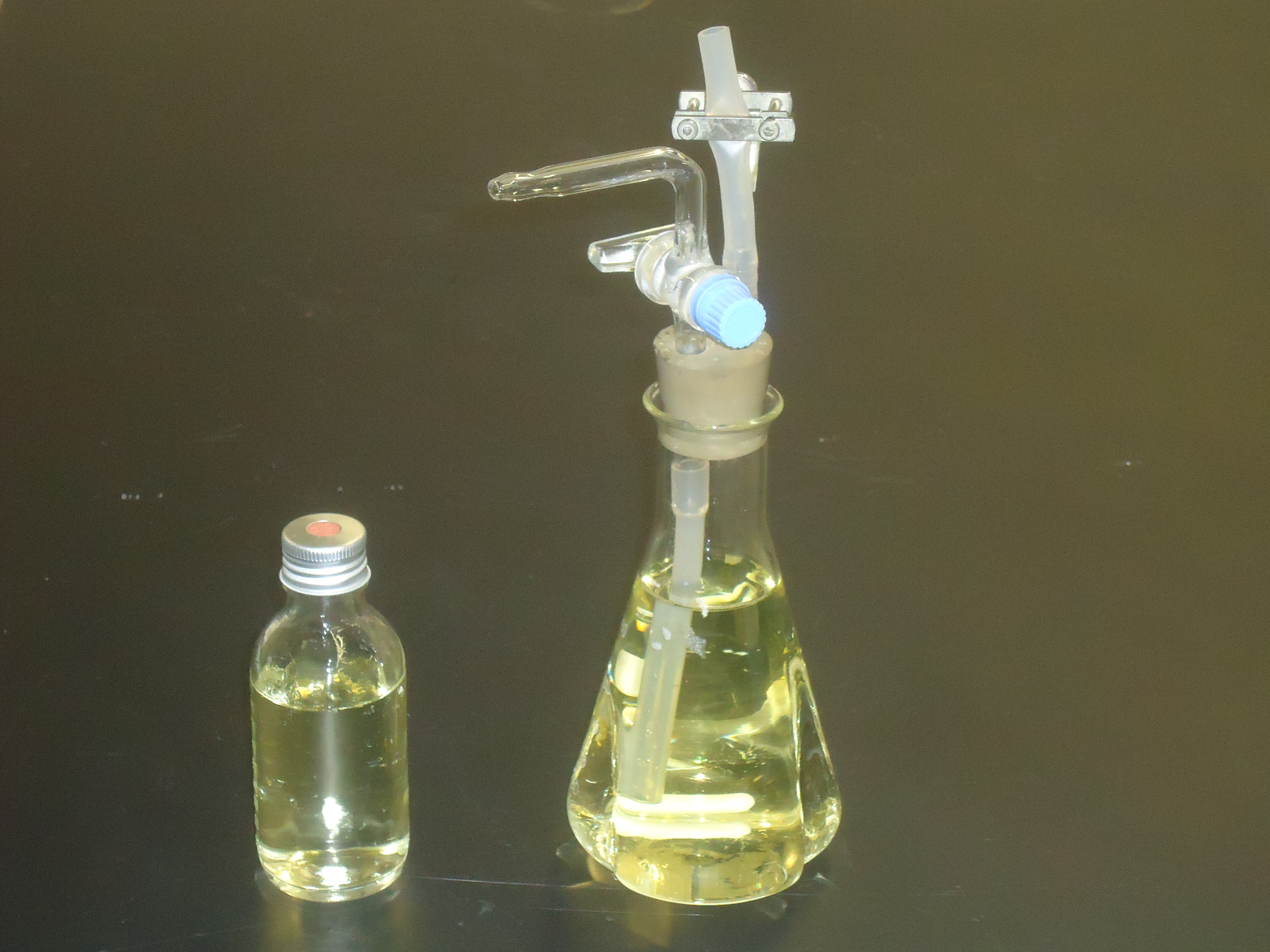


**Figure S1: Photograph showing the flasks used for anaerobic (*left*) and micro-aerobic (*right*) conversions of xylose.**
