# Supplementary figures and images for: Comparison of Scheffersomyces stipitis strains CBS 5773 and CBS 6054 with regard to their xylose metabolism: implications for xylose fermentation
Source: Microbiologyopen. 2012 Mar;1(1):64–70. doi: 10.1002/mbo3.5 (PMC3426399; doi:10.1002/mbo3.5)

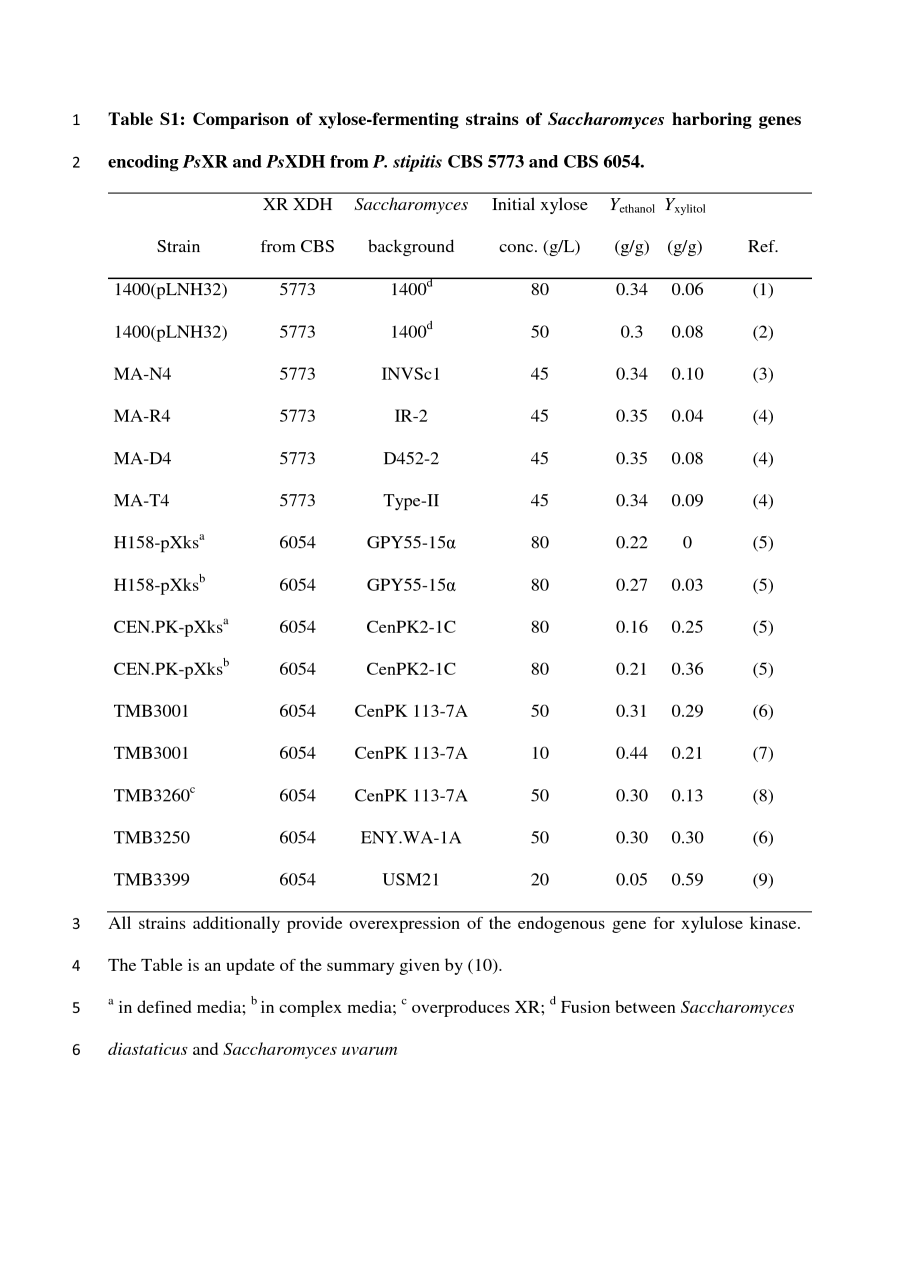

Supplement: Supplementary file 3 [file mbo30001-0064-SD3.png]
